# Toward dynamic tongue diagnosis: a conceptual framework for temporal phenotyping

**DOI:** 10.3389/fmed.2026.1888509

**Published:** 2026-08-25

**Authors:** Tianhao Wang, Jian Ren

**Affiliations:** Department of Traditional Chinese Medicine Diagnostics, Shandong University of Traditional Chinese Medicine, Jinan, Shandong, China

**Keywords:** digital health, reproducibility, telemedicine, temporal phenotyping, tongue diagnosis, video analysis

## Abstract

Digital tongue diagnosis has advanced considerably through image-based feature extraction and machine learning, yet its dominant analytical paradigm remains anchored in static, single-frame examination—an approach that inherently discards temporal information generated during the examination process, including stabilization dynamics, fluctuation patterns, and short-term physiological responsiveness. In this Perspective, we argue for a reorientation from static feature categorization towards temporal phenotyping. We propose a four-component analytical framework comprising: (i) standardized video acquisition with minimum technical specifications for clinical feasibility; (ii) temporal event alignment anchored to physiologically defined reference points such as protrusion onset and maximal extension; (iii) steady-state window identification using CIELAB ΔE-based criteria to isolate analytically meaningful intervals; and (iv) dynamic feature representation that distinguishes quantitative temporal metrics from clinically interpretable phenotypes. We further delineate the evidentiary requirements—biological plausibility, measurement reproducibility, analytical validity, and clinical utility—necessary to substantiate this paradigm shift, and outline a staged validation pathway from controlled laboratory studies to prospective clinical evaluation. Potential applications include longitudinal health monitoring, individualised assessment, and telemedicine integration. Despite substantial translational barriers, we contend that a temporally informed framework offers a more comprehensive and clinically meaningful foundation for next-generation digital tongue diagnosis systems.

## Introduction

Tongue examination has historically occupied a central role in traditional Chinese medicine (TCM), serving as a window into systemic physiological states. In contemporary research, digital methodologies have progressively refined this ancient practice by converting visible tongue manifestations into quantifiable data streams (1). The confluence of advanced imaging systems, colour calibration algorithms, automated segmentation techniques, and supervised machine learning has enabled investigators to extract and quantify tongue attributes—encompassing chromatic properties, coating distribution, surface fissuration, textural heterogeneity, and morphological configuration—with substantially greater consistency than unaided human observation (2–4).

Despite this trajectory of improvement, the vast majority of digital tongue studies continue to operate within a fundamentally static analytical paradigm. Standard practice typically involves selecting a single representative image from each examination session, extracting predefined features, and using them to train classification models. Even when several images are captured sequentially, the analysis invariably collapses this temporal sequence into a single representative snapshot (1). While such an approach has proven valuable for standardisation purposes, it introduces a conceptual constraint: the tongue is treated as a fixed visual object captured at an arbitrary instant, rather than as a biologically dynamic surface undergoing continuous change during the period of observation.

This constraint has practical consequences. When protruded for examination, the tongue undergoes a cascade of physiological processes including muscular stabilisation, moisture redistribution across its surface, transient changes in local perfusion, and subtle positional adjustments—all of which may influence its visible appearance (5). Some of this variation undoubtedly constitutes artifactual noise, while other components may harbour meaningful biological information. A static analysis framework cannot discriminate between these possibilities, nor can it characterise the temporal trajectory through which a tongue phenotype develops and stabilises during an examination sequence.

Moreover, the static model is fundamentally misaligned with clinical contexts involving serial monitoring. In chronic disease management and longitudinal follow-up, clinical value derives precisely from detecting change over time—whether a patient’s condition is improving, deteriorating, or remaining stable (6). Single-frame analyses, by definition, are poorly equipped to address such questions.

For these reasons, we propose that the field’s next developmental frontier is not incremental refinement of static classification performance, but a conceptual migration towards dynamic tongue diagnosis. We define this term as an analytical approach in which tongue manifestations are characterised as temporal phenotypes derived from standardised video-based observation, rather than from isolated captured frames. The objective is not to supplant existing static descriptors, but rather to situate them within a temporally structured framework capable of capturing how tongue features emerge, undergo stabilisation, and exhibit variation across the examination interval (7).

The present Perspective first examines the limitations that make a purely static categorisation insufficient as the field’s dominant paradigm. We subsequently introduce a conceptual framework for dynamic tongue diagnosis structured around four core components. We then outline the evidentiary architecture required to validate this approach, and conclude by examining the clinical opportunities, implementation barriers, and research priorities that will shape its future development.

## Why static categorisation is no longer sufficient

The static paradigm has constituted an indispensable foundation for digital tongue diagnostics. It has enabled standardised image-based workflows, facilitated systematic feature extraction pipelines, and supported the development of classification systems that exceed the reproducibility of subjective clinical judgement (6, 8). However, as the discipline matures, several inherent constraints of static categorisation have become increasingly consequential—constraints that are not merely technical in nature, but reflect a more fundamental incompatibility between a time-dependent biological observation and a temporally compressed analytical model.

### The frame-selection problem

Even under rigorously controlled imaging environments, the captured appearance of the tongue at any given moment may vary according to the precise timing of image acquisition, the degree of protrusion achieved, momentary muscular tension, the current state of salivary distribution, and transient specular reflections (9, 10). A single image therefore represents merely one point within a brief but continuous observational process. If the selected frame fails to reflect the characteristic phenotype, the entire downstream analysis inherits this sampling bias. Empirical studies of tongue image variability—though still limited—have reported non-negligible differences across consecutive frames in colour and texture metrics (10), underscoring the need for a more robust sampling strategy.

### Inability to distinguish persistent features from transient perturbations

Features that appear visually prominent within one frame may attenuate or disappear in adjacent temporal moments, whereas other characteristics may become evident only after the tongue achieves a more settled positional state. Static analysis conflates stable, physiologically meaningful manifestations with momentary artefacts. This becomes particularly problematic when the research objective extends beyond visual labelling towards understanding the reliability and biological significance of observed signals (11, 12). For example, subtle changes in tongue colour or coating distribution that appear only transiently during sustained protrusion might reflect autonomic or vascular adjustments, yet they would be entirely missed by a single-frame approach.

### Unsuitability for within-person longitudinal monitoring

Clinically relevant questions frequently concern temporal trajectories: the direction and rate of change in patient status, the presence or absence of treatment responsiveness, or the distinction between stable and labile physiological states (7). These questions are inherently unanswerable through isolated cross-sectional measurements. A temporally aware framework aligns more naturally with longitudinal clinical medicine by enabling phenotype characterisation in terms of stability, variability, and trajectory—dimensions that single-frame analysis cannot access.

### Conceptual constraint on the field’s evolution

Finally, the structural dominance of static image analysis has arguably constrained the field’s conceptual evolution. Research priorities organised around static classification tend to emphasise technical optimisation—improving segmentation precision, refining feature extraction, advancing model accuracy (13)—while leaving a more fundamental question underexamined: what constitutes the appropriate unit of observation for digital tongue diagnosis? If the clinical act of tongue examination inherently unfolds over time, the analytically appropriate unit may well be a structured temporal observation rather than a discretised frame.

**Figure 1:**
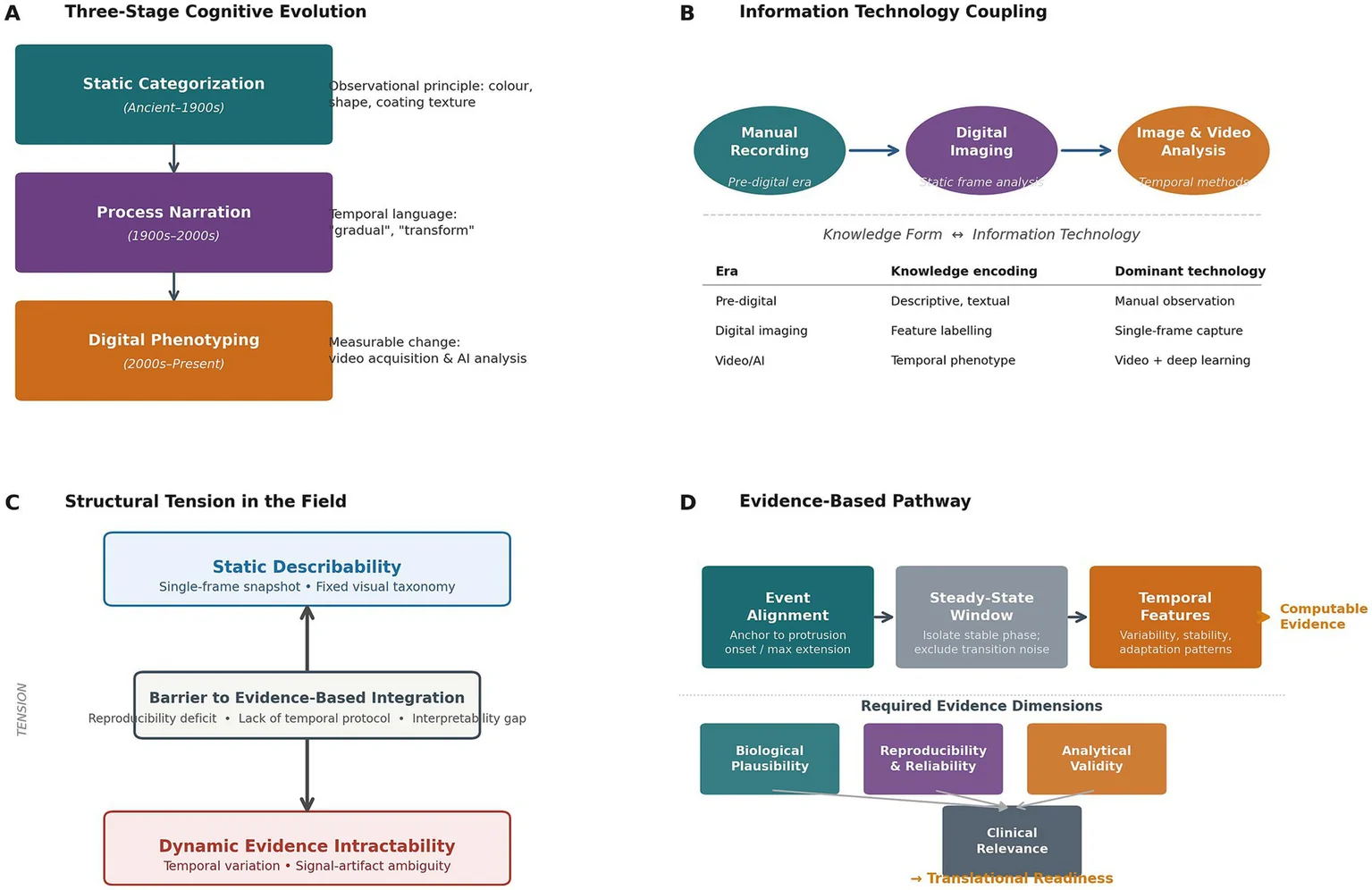
Evolution of Tongue Diagnosis Cognitive Paradigm Panel **D** illustrates the evidence-based pathway for validating dynamic tongue phenotyping, requiring four essential dimensions: biological plausibility (linking temporal variation to physiological mechanisms such as local perfusion dynamics, autonomic tone, and muscular tension), reproducibility and reliability (ensuring consistent measurement across recordings, sessions, and sites through operational transparency), analytical validity (distinguishing physiologically meaningful temporal signals from acquisition artefacts including motion blur and specular reflection), and clinical relevance (demonstrating utility in longitudinal monitoring and treatment response assessment). This evidentiary architecture underpins the transition from static categorisation towards dynamic phenotyping. Panels **A–C** contextualise the field’s evolution and the structural tensions between static describability and dynamic evidence intractability that necessitate this evidence-based approach.

These observations do not constitute a rejection of static methods, which retain genuine utility across many contexts. Rather, they establish the case for situating static descriptors within a broader temporal framework—a reorientation that sets the conceptual stage for temporal phenotyping (see Figure 1).

## A conceptual framework for dynamic tongue diagnosis

Ethics statement: This study is a conceptual framework development and does not involve human subject’s research.

**Figure 2:**
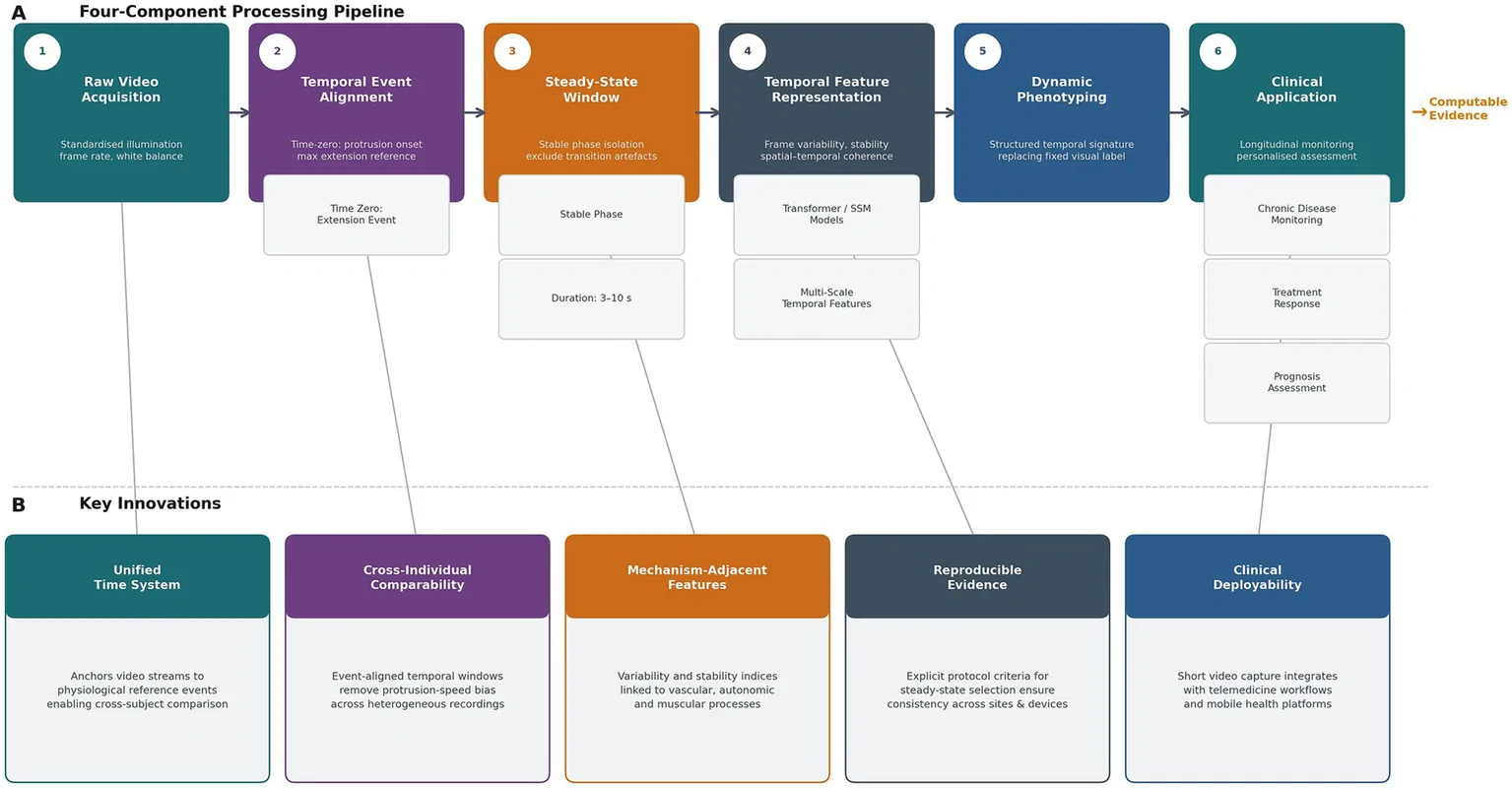
Evidence-based pathway for dynamic tongue diagnosis this framework illustrates the systematic implementation of dynamic tongue diagnosis through four core processing components and two output stages: (1) Raw video acquisition ensuring standardised illumination, frame rate, and white balance; (2) Temporal event alignment anchoring analysis to physiological reference points such as protrusion onset and maximal extension; (3) Steady-state window identification (candidate duration 3–10 s) isolating analytically meaningful intervals while excluding transition artefacts; (4) Temporal feature representation capturing inter-frame variability, stability indices, and spatial–temporal coherence; (5) Dynamic phenotyping generating structured temporal signatures encompassing appearance, stability, variability, and responsiveness; and (6) Clinical applications including longitudinal monitoring, treatment response assessment, and prognosis evaluation. Five key innovations enable this pathway: Unified time system establishing cross-subject comparability through event-aligned temporal windows; mechanism-adjacent features linking temporal patterns to vascular, autonomic, and muscular processes; reproducible evidence through explicit protocol criteria for steady-state selection; and clinical deployability via short video capture integrated with telemedicine workflows. This evidence-based framework transforms raw video data into computable evidence for dynamic tongue diagnosis.

The transition from static image analysis to temporal phenotyping requires more than a simple substitution of video sequences for still images. Raw video data, absent a principled analytical structure, does not inherently yield interpretable temporal information (1). What is required is a systematic framework that transforms tongue motion and appearance data into reproducible, meaningful temporal representations. We propose that this framework comprises four interdependent components (Figure 2).

### Standardised video acquisition

The foundation of dynamic tongue diagnosis rests upon standardised video acquisition. The fidelity and comparability of temporal signals are highly sensitive to acquisition conditions, which directly influence the tongue’s observed appearance throughout the recording interval. Parameters requiring systematic control include illumination spectrum and intensity, camera positioning and angular configuration, shooting distance, frame rate, spatial resolution, white balance settings, and environmental background consistency (5, 8, 14).

Equally important are the procedural instructions provided to subjects, governing tongue protrusion mechanics, maintenance duration, and management of breathing and swallowing movements. The collective influence of these variables on temporal tongue appearance underscores the necessity of treating video acquisition as a formal measurement protocol rather than an incidental recording step. Standardised acquisition is the prerequisite for any subsequent cross-individual, cross-session, cross-device, or cross-site comparison of temporal features.

To facilitate practical implementation, we propose minimum technical specifications as initial guidelines: a frame rate of at least 30 fps, a spatial resolution of at least 1,280 × 720 pixels, and a recording duration of at least 15 s to allow for a stable window and sufficient buffer. These values are intended as starting points for protocol development and should be empirically validated in future studies. The integration of standardised video acquisition with real-time processing pipelines has been demonstrated in other safety-critical domains—for example, road anomaly detection systems that combine standardised camera mounting protocols with temporal alignment procedures for embedded edge deployment (15)—providing practical engineering precedents for the standardisation and temporal alignment requirements of dynamic tongue diagnosis.

### Temporal event alignment

The second component addresses a fundamental challenge absent from static image analysis: the internal temporal heterogeneity of tongue examination videos (16). The examination sequence encompasses distinct phases—initial protrusion into the field of view, progressive forward extension, achievement of maximal or near-maximal displacement, maintenance of a stable position, and eventual retraction or positional drift. These phases are not analytically equivalent; frames captured during transition are not directly comparable to those obtained during stable maintenance.

Temporal event alignment resolves this heterogeneity by anchoring analysis to physiologically or procedurally defined reference points—such as protrusion onset, maximal extension, or the initiation of relative stability—rather than to absolute frame indices. This procedure reduces temporal misregistration, rendering between-subject comparisons more analytically defensible. The approach is conceptually analogous to event-centred analysis methodologies employed in other biomedical time-series domains, where comparison is organised around homologous temporal landmarks rather than raw chronological positions.

### Steady-state window identification

The third component recognises that dynamic observation does not imply uniform informational value across the recording interval. In most examination settings, the analytically most productive segment is a temporally delimited interval during which the tongue has achieved a relatively stable position and the disruptive influence of transition-related movement has subsided (10). Identifying and isolating this steady-state window enables reproducible measurement while reducing susceptibility to motion blur, unstable extension effort, variable angular positioning, and transient surface reflections that characteristically affect early and late portions of the recording.

The steady-state window functions as a standardised temporal segment suitable for both conventional static feature extraction and genuinely dynamic temporal descriptors, thereby bridging traditional analytical approaches with temporally informed methods. Selection criteria for this window should be explicit and reproducible, even if the precise window duration varies across recordings.

As a concrete operationalisation, we propose a candidate steady-state criterion (to be empirically calibrated in future protocol validation studies): window onset defined as the first frame after maximal extension where the mean inter-frame CIELAB ΔE across the tongue region falls below a threshold of 2.0 for at least 5 consecutive frames (approximately 0.17 s at 30 fps), indicating that gross positional and colorimetric change has subsided. The value of ΔE = 2.0 is derived from colour-difference perception thresholds commonly used in imaging applications (17); its applicability to tongue tissues requires validation. Window termination is defined by sustained ΔE increase above this threshold, signifying retraction onset or positional drift. The suggested 3–10 s window reflects two considerations: the lower bound (3 s) allows time for muscular stabilisation and initial moisture redistribution to settle, based on general muscle fatigue and oral moisture equilibration times reported in physiological studies (18); the upper bound (10 s) limits fatigue-related confounds and maintains clinical practicality. These parameters should be refined with empirical data.

To illustrate the conceptual trajectory of ΔE during a typical examination: upon maximal protrusion, ΔE values between consecutive frames are initially elevated (typically >3.0) due to gross positional adjustment and muscular settling. Over the subsequent 2–3 s, ΔE declines progressively as the tongue stabilises, crossing the proposed threshold of 2.0 to mark the onset of the steady-state window. During the steady-state interval, ΔE fluctuates within a narrow range (approximately 1.0–2.0), reflecting subtle physiological variations such as local perfusion dynamics and moisture redistribution. As fatigue or discomfort prompts retraction, ΔE rises again above the threshold, marking window termination. This three-phase trajectory—elevated transition, stable plateau, and rising retraction—provides an intuitive visualisation of the steady-state identification logic that future empirical studies should validate with actual tongue video data. The identification of such steady-state windows in medical video streams can be further informed by temporal modelling approaches developed for related domains—for example, multi-head Transformer architectures with adaptive loss functions have been successfully employed to capture temporal dependencies in facial landmark sequences for fatigue detection (19), offering methodological insights for modelling the temporal evolution of tongue position and surface features during sustained protrusion.

It is important to clarify that the signal-versus-artefact distinction is not inherent to any single physiological process but depends on temporal phase and analytical context. Salivary redistribution during the transition phase (protrusion, repositioning) constitutes artefact because it reflects postural adjustment rather than steady-state physiology; the same process operating within the steady-state window, however, may generate informative fluctuations reflecting autonomic regulation of salivary flow. Similarly, muscular fatigue during the transition phase is a source of positional instability to be excluded, whereas subtle fatigue-related changes in tissue appearance during the steady-state phase may carry diagnostic information. The framework thus discriminates signal from artefact not by process identity but by temporal context and magnitude: variations within the steady-state window that exceed measurement noise thresholds and co-vary with physiological parameters are candidate signals, while transition-phase perturbations are treated as artefact regardless of their underlying mechanism.

### Dynamic feature representation

The fourth component constitutes the analytical core of the framework: dynamic feature representation. Once videos have been standardised, temporally aligned, and segmented into informative windows, the task becomes defining features that capture meaningful temporal structure rather than merely replicating static descriptors at multiple time points (11, 20).

Candidate dynamic features encompass inter-frame variability metrics, rates of feature change across the observation interval, temporal stability indices, regional colour and texture fluctuation patterns, spatial–temporal coherence measures, and responsiveness or adaptation patterns. These features characterise behavioural properties across an interval—how stable, variable, or responsive tongue manifestations are under controlled conditions—rather than snapshot states at discrete moments (21).

To support computationally efficient extraction of these dynamic features in clinical settings, lightweight video processing architectures offer relevant methodological precedents. For instance, cascaded feature reweighting with complementary attention mechanisms has been shown to achieve robust performance with minimal computational overhead in real-time video analysis tasks (22), suggesting that similar efficiency gains may be achievable for tongue video feature extraction. Furthermore, for detecting subtle temporal variations in tongue surface texture and coating characteristics—which often present with low contrast against the surrounding tissue—multiscale edge-aware learning with hybrid attention mechanisms provides a promising approach for enhancing boundary delineation and fluctuation extraction (23). These computer vision methodologies collectively strengthen the technical feasibility of deploying dynamic feature extraction in resource-constrained clinical environments.

To avoid conceptual ambiguity, we distinguish between dynamic features (quantitative measures computed directly from video sequences, such as ΔE trajectory, coefficient of variation of colour channels, or rate of change of coating area) and temporal phenotypes (higher-level, clinically interpretable summaries derived from these features, such as “rapidly stabilising type,” “persistently fluctuating type,” or “slow-adapting type”). This hierarchical structure facilitates both computational reproducibility and clinical interpretation.

### Integration: from video to temporal phenotype

Taken together, these four components constitute an analytical pipeline that fundamentally reconceptualises the unit of tongue diagnosis. Standardised acquisition ensures data comparability; temporal alignment ensures correspondence across subjects and sessions; steady-state identification isolates analytically meaningful intervals; and dynamic feature representation converts these intervals into quantifiable temporal phenotypes (1, 2). The resulting output is not merely an enriched dataset but a qualitatively different unit of analysis: a structured temporal tongue signature that encompasses information about appearance, stability, variability, and responsiveness within a single integrated representation.

It is important to delineate what this framework inherits from existing paradigms and what is specific to tongue diagnosis. The concept of temporal event alignment borrows from event-related analysis in video-physiology (e.g., PhysFormer’s use of temporal difference transforms (16)), and the broader notion of digital phenotyping from continuous behavioural measurement is well established (24, 25). However, tongue diagnosis presents unique characteristics that necessitate domain-specific adaptation: (1) the tongue is a directly observable, highly vascularised muscular surface whose appearance integrates vascular, autonomic, and muscular dynamics on a single organ—unlike facial video-physiology where cardiovascular signals must be recovered through subtle chromatic variation; (2) the controlled protrusion protocol creates a standardised physiological perturbation with a defined onset, enabling temporal alignment that is not available in passive observational phenotyping; (3) the TCM diagnostic tradition has long recognised temporal qualities of tongue examination (how the tongue “settles,” the speed and manner of extension), providing a conceptual precedent for temporal analysis that has no parallel in general digital phenotyping. These features distinguish dynamic tongue diagnosis from direct application of existing video-based physiological measurement frameworks.

## Building the evidentiary foundation for dynamic tongue phenotyping

### Comparative analysis with existing approaches

To position the proposed temporal phenotyping framework within the broader landscape of computational tongue diagnosis and medical video analysis, we provide a theoretical comparison with three major categories of existing methods: (1) static image-based CNN classifiers, (2) recurrent neural network approaches for sequential data, and (3) emerging video transformer architectures.

#### Static CNN-based models

Conventional deep learning approaches for tongue diagnosis predominantly employ convolutional neural networks (CNNs) such as ResNet, EfficientNet, or custom architectures to extract spatial features from single tongue images (1, 2, 13, 26, 27). These models excel at recognising morphological patterns—colour distribution, coating texture, fissure shapes—but are fundamentally limited to static representations. Recent comprehensive reviews have documented the extensive application of CNNs and their variants in tongue image classification, segmentation, and disease prediction tasks (26, 27). However, these models cannot capture temporal dynamics such as stabilisation trajectories, moisture redistribution patterns, or subtle colour shifts during sustained protrusion. While static models achieve high classification accuracy for well-defined phenotypes, they lack the temporal resolution necessary to distinguish between stable physiological states and transient artefacts, limiting their utility for longitudinal monitoring and dynamic assessment.

#### Recurrent neural networks (LSTM/GRU)

Sequential models such as Long Short-Term Memory (LSTM) and Gated Recurrent Units (GRU) can process time-series data and capture temporal dependencies across frames. In principle, these architectures could model tongue appearance changes over time. In the broader medical video analysis literature, CNN-LSTM hybrids have been successfully applied to echocardiographic video classification (28), lung ultrasound video diagnosis (29), and breast nodule classification from ultrasound videos (30), demonstrating the value of combining spatial feature extraction with temporal sequence modelling. However, RNN-based approaches face several challenges in medical video contexts: (i) vanishing gradients limit their ability to capture long-range temporal dependencies; (ii) sequential processing creates computational bottlenecks for high-frame-rate video analysis; and (iii) they struggle with aligning heterogeneous temporal events (e.g., protrusion onset, steady-state onset) across subjects with varying movement speeds. Our framework addresses these limitations through event-aligned temporal windows rather than raw frame sequences, enabling cross-subject comparability independent of protrusion velocity.

#### Video transformers and temporal clustering

Recent advances in video understanding have introduced transformer-based architectures capable of modelling spatio-temporal relationships across video frames. As comprehensively surveyed in (31), models such as TimeSformer and the Video Swin Transformer (32) have demonstrated impressive performance in action recognition and have been increasingly applied to medical video analysis. These models leverage self-attention mechanisms to capture long-range dependencies across both spatial and temporal dimensions. However, their application to tongue diagnosis presents unique challenges: (i) medical video datasets are typically small-scale due to annotation costs and privacy constraints, limiting the data-hungry transformer architectures; (ii) tongue videos contain domain-specific temporal structures (protrusion, stabilisation, retraction) that require specialised alignment rather than generic temporal attention; and (iii) clinical interpretability demands explicit feature definitions tied to physiological mechanisms, which black-box transformer representations may not provide.

Beyond these end-to-end learning approaches, temporal clustering methods have been explored for segmenting medical video streams into meaningful phases—for example, in behaviour time series analysis (33) and in general medical video understanding tasks (34). These methods aim to partition video sequences into semantically coherent temporal segments without relying on predefined temporal labels. Our steady-state window identification component shares conceptual similarities with such approaches but differs in its explicit physiological grounding: rather than discovering temporal boundaries purely from data-driven clustering, we anchor segmentation to physiologically defined events (protrusion onset, maximal extension) and empirically calibrated stability criteria.

The key distinction of our temporal phenotyping framework lies in its emphasis on structured temporal analysis rather than end-to-end prediction. Rather than replacing existing classifiers, our framework provides a principled preprocessing pipeline—standardised acquisition, event alignment, steady-state isolation—that ensures input data quality and temporal consistency. This modular design allows integration with various downstream classifiers (CNNs, RNNs, transformers) while addressing the fundamental challenge of temporal heterogeneity in tongue examination videos.

## Rationale for benchmark selection

The evaluation of dynamic tongue diagnosis frameworks requires careful selection of performance benchmarks that reflect both technical capabilities and clinical utility. We propose three primary evaluation dimensions, each addressing a distinct aspect of system performance:

### Computational complexity

Clinical deployment of tongue diagnosis systems often occurs in resource-constrained environments—community clinics, mobile health platforms, or telemedicine settings with limited computational infrastructure. Evaluating computational complexity (measured in FLOPs, inference time, and memory footprint) is essential to ensure that temporal analysis pipelines remain feasible for real-world deployment. A framework requiring GPU-level computation may achieve superior accuracy in controlled settings but remains impractical for point-of-care applications. By explicitly assessing computational requirements, we enable informed trade-offs between analytical sophistication and deployability. Recent work in medical video analysis has increasingly emphasised the importance of computational efficiency, with studies demonstrating that lightweight architectures can achieve competitive performance with substantially lower computational costs (34). The trade-off between model complexity and clinical deployability is particularly acute in video-based tongue diagnosis, where frame-rate processing and real-time feedback may be required in telemedicine consultations.

### Temporal modeling capability

The core innovation of dynamic tongue diagnosis lies in its ability to capture and represent temporal phenomena—stabilisation trajectories, fluctuation patterns, and responsiveness metrics. Evaluation must therefore assess not merely classification accuracy but the fidelity of temporal representation. Relevant metrics include: (i) temporal resolution (minimum detectable change duration); (ii) stability indices (variance of features within steady-state windows); (iii) trajectory characterisation (ability to model non-linear temporal patterns); and (iv) event detection accuracy (precision in identifying protrusion onset, steady-state onset, and retraction). These metrics distinguish frameworks that merely process video frames from those that genuinely model temporal dynamics. In the broader medical video analysis literature, establishing such temporal benchmarks is critical for moving beyond static accuracy towards clinically meaningful temporal reasoning (31, 34).

### Robustness in medical video contexts

Medical videos present unique challenges distinct from consumer video applications: illumination variability, motion artefacts from patient movement, limited annotated datasets, and stringent requirements for reliability and interpretability. Robustness evaluation should assess performance under controlled perturbation of nuisance variables (illumination changes, partial occlusions, variable protrusion speeds) and consistency across diverse populations and recording conditions. Unlike generic video analysis tasks where accuracy on benchmark datasets suffices, medical applications demand demonstrated reliability across heterogeneous real-world conditions. Frameworks that achieve high accuracy on curated datasets but degrade under minor environmental variations lack clinical utility. Recent empirical evaluations of video models in clinical screening contexts have highlighted the substantial performance gaps that emerge when models are tested under realistic corruption and distribution shifts (31, 34), underscoring the necessity of robustness as a primary evaluation dimension.

These three dimensions—computational complexity, temporal modelling capability, and robustness—collectively address the practical feasibility, technical soundness, and clinical applicability of dynamic tongue diagnosis systems. Future benchmarking efforts should develop standardised evaluation protocols incorporating these criteria, enabling systematic comparison across frameworks and accelerating translational progress.

### Biological plausibility

Temporal variation in tongue features acquires evidential significance only when it can be plausibly linked to known or hypothesised physiological mechanisms. The tongue is a highly vascularised, mechanically active, moisture-sensitive tissue whose surface characteristics may respond to fluctuations in local perfusion dynamics, autonomic nervous system tone, muscular tension and fatigue, salivary distribution, respiratory patterns, and transient environmental exposures during examination (12, 35). These physiological processes collectively provide a rationale for the existence of structured temporal variation and for the potential informational content of such variation.

The biological plausibility argument rests on mechanisms operating at distinct timescales, only some of which are directly relevant to the examination window. Processes active within seconds to tens of seconds include local perfusion dynamics, autonomic vascular regulation, muscular stabilisation and fatigue, salivary redistribution, and transient specular reflections from moisture changes—all directly observable during a standard examination. Processes operating over minutes to hours, such as meal-related salivary composition shifts and postural perfusion adaptation, are contextual but not directly captured within a single examination session. Slower processes operating over hours to days, including circadian microbiota variation (12) and hormonal influences on mucosal vascularity, provide background physiological state rather than within-examination dynamics. The temporal phenotyping framework specifically targets the first tier—processes plausibly active within the seconds-to-tens-of-seconds examination window. Slower processes establish the background state that determines the starting point of any temporal trajectory but do not themselves generate within-examination temporal variation. This distinction clarifies why mechanisms such as diurnal microbiota rhythms (12), while relevant to between-session variation, do not directly support the existence of within-examination temporal signals.

Although direct demonstration awaits systematic video-based data collection, a candidate temporal trajectory can be conceptually illustrated: during sustained protrusion, a highly vascularised tongue region may exhibit a gradual increase in red-channel intensity over the initial 3–5 s as local perfusion adapts to postural demand, followed by plateau as autonomic regulation stabilises—yielding a characteristic rise-to-plateau curve (for illustrative purposes only). Whether such trajectories are consistently detectable, reproducible, and diagnostically informative remains an open empirical question that the present framework is designed to address, not a claim it assumes as established.

However, biological plausibility demands empirical verification beyond theoretical inference. Future research should examine whether specific dynamic features co-vary with independently measurable physiological parameters—for instance, whether temporal colour intensity changes correlate with markers of microcirculatory behaviour, or whether stability-related features associate with indices of postural control or muscular fatigue during sustained protrusion. Such investigations would anchor dynamic phenotypes in measurable biology rather than leaving them as abstract computational constructs.

A critical open question concerns the diagnostic specificity of these temporal signals. Many physiological states—stress, fatigue, postural changes—can affect local perfusion and muscular tension within seconds, yet these effects may not be specific to any particular TCM syndrome or disease category. The framework does not presuppose that all temporal variation is diagnostically meaningful; rather, it posits that the pattern of variation (e.g., rapid vs. slow stabilisation, high vs. low lability) may carry information about an individual’s physiological regulatory capacity. Establishing specificity will require systematic comparative studies across diagnostic groups, and this remains a key task for future empirical research. Physiological interpretability is a necessary but not sufficient condition for clinical utility.

### Reproducibility and measurement reliability

A dynamic tongue feature lacks practical value in the absence of consistent measurability across repeated recordings, examination sessions, hardware platforms, and clinical sites (6, 8). Reproducibility assumes heightened importance in temporal phenotyping because the temporal dimension multiplies the potential sources of variability. Minor differences in instruction delivery, temporal alignment procedures, illumination stability, recording duration, or motion control protocols may all systematically influence resulting feature values.

Multiple reproducibility strata warrant assessment: within-session repeatability (stability across brief repeated recordings under identical conditions), between-session reproducibility (consistency across separate visits under presumed similar physiological states), and cross-device and cross-site generalisability (robustness under realistic variations in hardware and implementation environments). Reproducibility is further contingent upon operational transparency—clear, detailed specification of alignment procedures, window selection criteria, exclusion rules, and feature computation methods—without which apparent agreement may merely reflect local analytic conventions rather than genuine methodological stability (8).

### Analytical validity and artefact discrimination

Perhaps the most technically demanding evidential requirement is establishing analytical validity, specifically the capacity to distinguish physiologically meaningful temporal signals from acquisition artefacts (10, 16). Video-based signals are inherently vulnerable to confounding by motion blur, variable protrusion effort, specular reflection dynamics, illumination drift, camera auto-adjustment processes, and segmentation instability. Failure to address these sources of artefact systematically risks yielding models that identify apparent temporal features reflecting measurement error rather than genuine biological phenotype.

Analytical validation should interrogate whether temporal features demonstrate sensitivity to artefact conditions, whether they remain stable under controlled perturbation of nuisance variables, whether they can be recovered across different preprocessing pipelines, and whether they preserve discriminative structure following quality control filtering. For a developing field, interpretable feature designs with transparent physiological rationales may ultimately prove more valuable than high-dimensional representations that achieve impressive predictive performance but resist biological interpretation.

## Clinical relevance and application specificity

Temporal phenotyping must ultimately be justified through demonstrated clinical value—specifically, through evidence that it addresses limitations that static assessment handles inadequately (7, 20, 36–38). Different clinical application contexts will generate different evidentiary demands: longitudinal monitoring applications require sensitivity to within-person change rather than cross-sectional classification performance; screening applications demand incremental value beyond static features and routine clinical data; mechanistic research applications prioritise interpretability and biological linkage over immediate predictive accuracy.

The temporal phenotyping framework is advantageous in this regard precisely because it encourages application-specific evidence generation rather than global claims about diagnostic superiority. By asking under which clinical conditions temporal information is most informative and actionable, investigators can focus methodological development on contexts where the added complexity of video acquisition and temporal analysis is genuinely justified.

### Staged research strategy

The evidence base for dynamic tongue phenotyping should be developed through staged methodological progression. Initial investigations should prioritise protocol development, signal characterisation, and reliability quantification under controlled laboratory conditions. Subsequent studies can explore biological correlations, compare alternative feature representations, and assess generalisability across diverse populations and recording environments. Later-phase research should evaluate clinical utility through prospective designs with pre-specified endpoints in clearly defined application contexts (6).

### Clinical opportunities and translational challenges

Validated temporal tongue phenotypes have the potential to generate clinical value exceeding that achievable through static image-based analysis—not through the novelty of video technology per se, but by enabling observational modalities more naturally aligned with monitoring, responsiveness assessment, and individualised characterisation. Simultaneously, the translational pathway from conceptual framework to clinical deployment presents substantial obstacles that require careful prospective consideration.

### Longitudinal monitoring and trajectory characterisation

The most immediately apparent clinical opportunity lies in longitudinal monitoring applications. Static tongue assessment is structurally limited as a monitoring instrument because its snapshot nature provides no information about within-person temporal dynamics (7). If temporal phenotypes can be reliably measured across examination sessions, they may enable tracking of within-person variation in physiological status, symptom evolution, or treatment responsiveness in a manner genuinely individualised to each patient’s baseline characteristics and characteristic patterns.

This potential is particularly relevant in chronic disease management, rehabilitation programmes, and post-treatment surveillance, where trends and fluctuations frequently carry greater clinical significance than isolated cross-sectional categorisations. A patient’s temporal tongue signature may demonstrate relative stability across one clinical phase while exhibiting increased lability during another, even in the absence of substantial changes in static morphology—a pattern of information that would be entirely invisible to snapshot-based assessment.

### Short-term physiological responsiveness assessment

A complementary opportunity involves characterising short-term physiological responsiveness. Dynamic tongue diagnosis, by capturing temporal behaviour within the examination window, may be suited to detecting transient changes associated with physiological adjustment processes, hydration states, autonomic responsiveness, therapeutic interventions, or stress exposure (35). This application is conceptually distinct from static appearance description; it asks not what the tongue looks like at a given moment, but how tongue features behave over a structured observational interval.

Responsiveness-oriented assessment may be particularly valuable in settings where treatment effects emerge subtly, early in the therapeutic course, or heterogeneously across patient populations. Temporal phenotypes might serve as candidate markers of early treatment response before structural changes become apparent, providing supplementary evidence about physiological adaptation that static assessment cannot access.

### Personalised and context-aware phenotyping

Dynamic characterisation also opens pathways towards more genuinely personalised phenotyping. Static classification frameworks compare individual observations against population-derived categorical norms, which can be valuable for standardisation but inevitably underrepresents individual-level variability (20). Temporal phenotyping, by characterising stability, fluctuation patterns, and responsiveness properties, enables a more granular individual phenotypic profile that may carry different clinical implications across individuals—even when static appearance appears similar.

This approach aligns with broader precision medicine trajectories, in which clinically actionable information increasingly emerges from personal baselines, longitudinal trajectories, and multi-modal integration rather than from single-point cross-sectional measurements alone (25).

### Telemedicine and digital health integration

Dynamic tongue diagnosis may demonstrate particular compatibility with telemedicine and consumer-facing digital health platforms (7). As mobile imaging and remote consultation services become more prevalent, standardised short-duration tongue video capture may offer reproducibility advantages over reliance on single self-photographed images. Video data permits post-hoc quality control assessment, enables extraction of usable steady-state intervals from variable-quality recordings, and reduces dependence on perfect single-frame timing—all advantages that would improve the reliability of remote tongue assessment in real-world conditions (8).

Looking beyond current applications, temporal phenotypes could eventually be incorporated into multimodal monitoring frameworks integrating diverse digital biomarkers—including physiological wearable data, patient-reported outcomes, and behavioural indicators—where tongue temporal dynamics might serve as complementary physiological state markers.

### Translational barriers

Despite these opportunities, translational implementation faces meaningful challenges. Protocol heterogeneity across clinical sites, variable device quality in consumer settings, the computational demands of temporal analysis pipelines, limited interpretability of complex temporal features to clinical users, and the absence of established regulatory pathways for this class of digital measurement collectively constitute significant barriers. Furthermore, the additional burden of video acquisition relative to single image capture requires that the incremental clinical value be clearly demonstrable to justify adoption. These barriers are not insurmountable but demand systematic prospective attention as the field develops.

## Conclusion

Digital tongue diagnosis has achieved substantial progress through the translation of traditional observational descriptors into computationally analysable feature sets. Yet its prevailing methodological paradigm remains fundamentally static, treating the tongue primarily as a morphologically fixed visual object rather than as a biologically active surface observed under temporally evolving conditions (1, 2). This Perspective argues that while the static framework has been historically productive, it is no longer adequate to capture the full informational potential embedded in tongue examination.

We have proposed a structured transition towards dynamic tongue diagnosis, organised around four core components: standardised video acquisition, temporal event alignment, steady-state window identification, and dynamic feature representation. This framework does not invalidate existing static descriptors, but recasts them as one layer within a more comprehensive temporal measurement architecture. In doing so, it redefines the fundamental unit of analysis from a discretised frame selected at an arbitrary instant to a purposefully structured temporal observation (1, 5).

**Key innovative contributions** of this framework can be summarised as follows:

1. **Temporal unit of analysis.** The framework reconceptualises the fundamental analytical unit from a single static image to a structured temporal observation encompassing stabilisation dynamics, fluctuation patterns, and responsiveness characteristics—a shift that enables the characterisation of tongue phenotypes in terms of stability, variability, and trajectory rather than static morphology alone.
2. **Physiologically anchored event alignment.** Unlike generic video analysis approaches that treat frames as a uniform sequence, our framework introduces alignment to physiologically defined reference points (protrusion onset, maximal extension, steady-state initiation), enabling cross-subject comparability independent of variable protrusion velocity—a capability that existing CNN, LSTM, and video transformer models do not provide.
3. **Operationalised steady-state criterion.** The framework provides a concrete, empirically testable criterion for steady-state window identification (ΔE < 2.0 for ≥5 consecutive frames), transforming an abstract conceptual need into a quantifiable protocol that can be validated, refined, and standardised across studies and clinical sites.
4. **Hierarchical feature-phenotype distinction.** By distinguishing between quantitative dynamic features (computational metrics) and interpretable temporal phenotypes (clinically meaningful summaries), the framework bridges the gap between computational reproducibility and clinical interpretability—addressing a limitation common to black-box deep learning approaches.
5. **Staged evidentiary pathway.** The framework delineates a four-dimensional evidence architecture (biological plausibility, reproducibility, analytical validity, clinical utility) with a staged research strategy, providing a roadmap for systematic validation that moves beyond isolated accuracy metrics toward clinically meaningful evaluation.

These contributions are conceptual in nature and await empirical validation through the staged research strategy outlined above. Future work should focus on: (i) prospective video data collection to empirically calibrate the proposed ΔE threshold and window duration parameters; (ii) quantitative comparisons against static baselines, CNN-LSTM hybrids, and Video Swin Transformer models to substantiate the framework’s incremental value; and (iii) clinical utility studies in defined application contexts such as longitudinal monitoring of chronic conditions or treatment response assessment.

The scientific legitimacy of this transition will depend entirely on rigorous evidence generation. Dynamic tongue phenotyping must demonstrate biological plausibility through physiological correlation studies, methodological reproducibility across devices and settings, analytical validity through artefact discrimination and interpretable feature design, and clinical utility through application-specific prospective evaluation. Development should proceed through staged methodological progression rather than premature emphasis on predictive performance (6, 35).

Should this transition be achieved with appropriate methodological rigour, tongue diagnosis stands to evolve from a descriptive, snapshot-based practice into a standardised, longitudinally informed, mechanistically grounded component of digital medicine. The field’s future trajectory depends not solely on achieving higher visual classification accuracy at isolated moments, but on developing the scientific and technical infrastructure to measure, validate, and apply what the tongue reveals over time (7, 20).

## Data Availability

The original contributions presented in the study are included in the article/supplementary material, further inquiries can be directed to the corresponding author.
